# Angiogenesis as Therapeutic Target in Metastatic Prostate Cancer – Narrowing the Gap Between Bench and Bedside

**DOI:** 10.3389/fimmu.2022.842038

**Published:** 2022-02-10

**Authors:** Antonio Giovanni Solimando, Charis Kalogirou, Markus Krebs

**Affiliations:** ^1^Department of Biomedical Sciences and Human Oncology, Section of Internal Medicine “G. Baccelli”, University of Bari Medical School, Bari, Italy; ^2^Medical Oncology Unit, Istituto di Ricovero e Cura a Carattere Scientifico (IRCCS) Istituto Tumori “Giovanni Paolo II”, Bari, Italy; ^3^Department of Urology and Pediatric Urology, University Hospital Würzburg, Würzburg, Germany; ^4^Comprehensive Cancer Center Mainfranken, University Hospital Würzburg, Würzburg, Germany

**Keywords:** Prostate adenocarcinoma, PCa, angiogenesis inhibitors, TKI, immunotherapy, tumor microenvironment, clinical trials, PSMA

## Abstract

Angiogenesis in metastatic castration-resistant prostate cancer (mCRPC) has been extensively investigated as a promising druggable biological process. Nonetheless, targeting angiogenesis has failed to impact overall survival (OS) in patients with mCRPC despite promising preclinical and early clinical data. This discrepancy prompted a literature review highlighting the tumor heterogeneity and biological context of Prostate Cancer (PCa). Narrowing the gap between the bench and bedside appears critical for developing novel therapeutic strategies. Searching clinicaltrials.gov for studies examining angiogenesis inhibition in patients with PCa resulted in n=20 trials with specific angiogenesis inhibitors currently recruiting (as of September 2021). Moreover, several other compounds with known anti-angiogenic properties – such as Metformin or Curcumin – are currently investigated. In general, angiogenesis-targeting strategies in PCa include biomarker-guided treatment stratification – as well as combinatorial approaches. Beyond established angiogenesis inhibitors, PCa therapies aiming at PSMA (Prostate Specific Membrane Antigen) hold the promise to have a substantial anti-angiogenic effect – due to PSMA´s abundant expression in tumor vasculature.

## Introduction

The biological context of angiogenesis and prostate cancer (PCa) inspired a plethora of research, specifically in metastatic PCa and more specifically in castration-resistant disease (CRPC), the clinical stage in which the majority of clinical trials on angiogenesis inhibition was performed (1). Metastatic PCa is an androgen-driven and -dependent cancer (2), with androgen deprivation therapy (ADT) being the primary treatment. Despite high response rates – practically 90% of patients initially respond to hormone therapy – the vast majority will end up relapsing (3) in a predictable and irreversible manner. There has been a fair amount of research to try to analyze the mechanisms of progression to CRPC, which is the lethal phenotype of metastatic PCa – and current evidence suggest a function of clonal selection and adaptation by androgen receptor (AR)-dependent and independent mechanisms (4).

Indeed, ADT together with next generation hormonal agents such as Abiraterone (5) and Enzalutamide (6) still represent the foundation of systemic PCa treatment. Beyond hormone therapy, approved chemotherapy regimens mainly consist of Docetaxel and Cabazitaxel as microtubule inhibitors (7–9). Regarding bone as a favorite localization of PCa metastasis (10–12), therapeutic (combination) approaches include Radium-223 (13). In recent years, PCa treatment has rapidly developed towards precision oncology by addressing two novel target pathways: DNA repair and Prostate-specific membrane antigen (PSMA)-related signaling. Regarding DNA repair, cancers with mutations in BRCA1/2 (Breast Cancer Associated Genes 1 and 2) can be treated with PARP (Poly-ADP-Ribose-Polymerase) inhibitors originally established in Ovarian Cancer (14, 15). For PSMA, strategies include radioligand therapy as a theragnostic approach performed by nuclear medicine specialists (16).

Beyond these established and approved cancer therapies, this review aims to address an obvious treatment gap – given the crucial role of angiogenesis for PCa development and progression. Despite this fundamental promise reflected by *in vitro* and preclinical evidence, phase III trials with angiogenesis inhibitors failed to meet clinical endpoints.

## Prostate Cancer and VEGF-Mediated Angiogenesis – Promises and Challenges

About 50 years ago, Folkman and colleagues highlighted the importance of angiogenesis and neovascularization for tumor growth – reasoning that targeting tumor blood vessels might prove beneficial for patients with cancer (17). Meanwhile, state-of-the-art techniques highlighted the crucial but not completely understood link between angiogenesis (endothelial cells) and tumor immunity (18). For PCa, histopathology pinpoints high micro-vessel density and increased VEGF (Vascular Endothelial Growth Factor) expression compared to non-neoplastic conditions. Moreover, VEGF levels are associated with higher tumor stages as well as advanced grading and plasma VEGF is increased in metastatic PCa versus localized disease (19–21). Higher VEGF expression evaluated by immunohistochemistry has also been associated with reduced disease-specific survival in patients with PCa (22). In addition, levels of urinary VEGF were associated with worse survival (23) and elevated plasma VEGF/sVCAM-1, a vascular cell adhesion molecule, correlated with worse outcome (24).

In principle, many drugs and angiogenic target structures known from other solid and hematological malignancies are available for PCa (25–30). As a consequence, clinical trials combined antiangiogenic agents with Taxanes in mCRPC (31); however, not a single drug combined with Docetaxel showed a statistically significant success in terms of outcome (32). Therefore, clinicians started trials in less symptomatic patients, investigating compounds as single agents. Unfortunately, all of these phase III trials with thousands of patients were collectively negative for OS – despite promising biological preclinical as well as promising phase II trials. Despite efforts studying more than 1,000 patients, the combination of Bevacizumab or Aflibercept with chemotherapy showed no improvement compared to chemotherapy alone (33, 34). Sunitinib as a single agent compared to prednisone showed no improvement, either (35).

Making it even worse, Lenalidomide treatment resulted in a sobering scenario (36): While effective in several hematologic conditions (37–40), combination treatment of patients with PCa (Lenalidomide + Docetaxel + Prednisone) led to a significantly worse OS compared to treatment with Docetaxel and Prednisone (36). Another surprising and quite sobering example is Cabozantinib, an oral inhibitor of Tyrosine Kinases including MET and VEGFR2, two major drivers of malignant progression in several neoplasia (41–47), which did not guarantee an OS advantage in patients with PCa (48). Indeed, Cabozantinib showed anti-angiogenic and antitumor effects in a wide range of preclinical tumor models (49–51), also blocking progression of PCa xenografts in soft tissue and bone (52–54). Additionally, Cabozantinib affected key actors of the bone niche – with reduction in osteoclasts and biphasic effects osteoblasts, while altering bone remodeling with increased volume in mice (55). MET and VEGFR2 cooperate to promote tumor survival, thereby boosting angiogenesis *via* improved tumor blood flow and improved oxygenation. Moreover, MET promotes migration and invasion, also facilitating the escape from hypoxic areas. Consequently, bone metastases are associated with high levels of MET expression. In specific, MET expression increased with androgen deprivation in preclinical models and with progression and metastasis in bone and lymph nodes (56). Promising early phase II trial results from bone scans upon combined Docetaxel and Cabozantinib treatment showed activity in 300 patients (48, 57). Soft tissue effects were also present, with objective response and significant progression-free survival (PFS) benefit (48). Improvement in pain and reduction of narcotics corroborated these initial results (58). These data were paralleled by a reduction of circulating tumor cells (57), while keeping activity in subjects heavily pretreated with Docetaxel, Abiraterone and/or Enzalutamide (48, 57). The lowest effective dose of these studies was 40 mg/day (59). Nevertheless, within phase III trial, Cabozantinib did not perform better than Prednisone (60). The dose and the stage of disease could have been the cause for this failure.

## Current Clinical Trials on Anti-Angiogenesis in Prostate Cancer

To determine the *status quo* of clinical trials investigating anti-angiogenesis in PCa, we performed a database research on clinicaltrials.gov. As of September 2021, a total sum of 866 actively recruiting interventional trials were registered for patients suffering from PCa. As outlined in **Table 1**, only a minority of clinical trials investigated the effects of angiogenesis inhibitors/Tyrosine kinase inhibitors. Specifically, we identified 20 clinical trials addressing angiogenesis inhibition. While some studies aim to identify predictive biomarkers for future clinical stratification in entity-independent trials (NCT02465060, NCT03878524), others combine angiogenesis inhibition with immune checkpoint blockade – e. g. CONTACT-02 trial investigating Cabozantinib in combination with Atezolizumab in patients with mCRPC (NCT04446117). Of note, other studies include patients in different stages, such as metastatic castration sensitive disease (CABIOS phase I trial, NCT04477512) and even localized disease in a neoadjuvant setting before Radical Prostatectomy (SPARC phase II trial, NCT03964337).

**Table 1:** Recruiting interventional trials examining anti-angiogenesis in prostate cancer (PCa) registered within clinicaltrials.gov database (December 2021).

Trial Identifier	Stage/Entity	Title/characteristics	Treatment	Comment
NCT01567800	PCa	Prostate Hypoxia FAZA	18F-FAZA	Hypoxia-specific PET tracer
NCT02465060	Advanced Cancer	MATCH screening trial; Phase II	(…), Sunitinib, (…)	Biomarker-driven Basket trial for various compounds
NCT02484404	Advanced solid tumors	Phase I/II	Combinations of Cediranib, Durvalumab and Olaparib	Cediranib: pan-VEGFR inhibitor
NCT02643667	Localized PCa	Phase I/II	Ibrutinib before Radical Prostatectomy	Ibrutinib: BTK inhibitor; Neoadjuvant setting
NCT03170960	Advanced solid tumors	Phase I/II	Cabozantinib ± Atezolizumab	
NCT03385655	PCa	Phase II	(…), Savolitinib, (…)	Biomarker-driven therapy stratification
NCT03556228	PCa and other malignancies	Phase I	VMD-928	VMD-928: TrkA inhibitor
NCT03845166	Advanced solid tumors	Phase I	XL092 AND Atezolizumab OR XL092 AND Avelumab	XL092: Tyrosine Kinase inhibitor (incl. VEGFR2)
NCT03866382	Rare genitourinary tumors	Phase II	Cabozantinib AND Nivolumab AND Ipilimumab	Metastatic Prostate Small Cell Neuroendocrine CA
NCT03878524	Advanced Cancer	SMMART; Phase I	(…), Bevacizumab, Cabozantinib, Sorafenib, Sunitinib, (…)	Biomarker-driven Basket trial for various compounds
NCT03964337	PCa before surgery	SPARC; Phase II	Neoadjuvant Cabozantinib	
NCT04159896	mCRPC	Phase II	ESK981 AND Nivolumab	ESK981: Pan-VEGFR/TIE2 inhibitor
NCT04446117	mCRPC	CONTACT-02; Phase III	Cabozantinib AND Atezolizumab	
NCT04477512	mCSPC	CABIOS; Phase I	Cabozantinib AND Abiraterone/Prednisone AND Nivolumab	
NCT04514484	Advanced Cancer AND HIV infection	Phase I	Cabozantinib AND Nivolumab	
NCT04521686	Advanced solid tumors with IDH1 mutations	Phase I	LY3410738	LY3410738: IDH1 inhibitor
NCT04631744	mCRPC	Phase II	Cabozantinib	
NCT04635059	PCa: biochemical recurrence	BLAST; Phase II	Pacritinib	Pacritinib: JAK/FLT3 inhibitor
NCT04742959	Advanced solid tumors	Phase I/II	TT-00420 ± Nab-Paclitaxel	TT-00420: Tyrosine Kinase inhibitor (incl. VEGFRs)
NCT04848337	Advanced/metastatic neuroendocrine PCa	PLANE-PC; Phase II	Lenvatinib AND Pembrolizumab	Lenvatinib: VEGFR inhibitor
**Further compounds with known anti-angiogenic properties**				
NCT02935205	CRPC	Phase I/II	Indomethacin AND Enzalutamide	
NCT00268476	mCSPC	STAMPEDE; Phase II/III	(…), Metformin, (…)	
NCT01864096	low-risk PCa under Active Surveillance	MAST; Phase III	Metformin	
NCT02064673	PCa after Radical Prostatectomy	Phase III	Curcumin	
NCT02176161	PCa after therapy and a high-risk setting	Phase II	Metformin	
NCT02804815	PCa and other malignancies after curative therapy	Phase III	Aspirin	
NCT03031821	PCa with indication for ADT	PRIME; Phase III	Metformin AND ADT	
NCT03535675	PCa: PSA recurrence after definitive treatment	Phase III	Muscadine Grape extract	Patient pre-selection according to genotype
NCT03769766	low-risk PC under Active Surveillance	Phase III	Curcumin	
NCT03819101	CRPC	PEACE-4; Phase III	Acetylsalicylic acid ± Atorvastatin	
NCT03899987	PCa before Radical Prostatectomy	Phase II	Aspirin AND Rintatolimod ± interferon-alpha 2b	
NCT04300855	PCa under Active Surveillance	Phase II	Green Tea Catechins (Sunphenon)	
NCT04519879	PCa: recurrent/therapy-naive	Phase III	White Button Mushroom extract	
NCT04536805	PCa: relapse in previously irradiated Prostate bed	REPAIRGETUGP16; Phase I/II	Metformin AND Radiation	
NCT04597359	PCa under Active Surveillance	Phase II	Green Tea Catechins	

Ctr, Control; CRPC, castration-resistant Prostate Cancer; CSPC, castration-sensitive Prostate Cancer; mCRPC, metastatic castration-resistant Prostate Cancer; mCSPC, metastatic castration-sensitive Prostate Cancer; ADT, Androgen deprivation therapy.

Beyond this relatively small number of trials directly aiming at tumor vessels, we found several studies investigating compounds known to have additional anti-angiogenic effects (bottom part of **Table 1**). Curcumin, Green Tea Catechins and Metformin were among the substances identified. For Metformin, a tumor suppressive role was shown in several cancer entities (61). Moreover, adjuvant Metformin intake was associated with improved outcome in Clear Cell Renal Cell Carcinoma patients treated with Tyrosine Kinase inhibitors in two independent cohorts (62, 63). One reason for this protective effect could be the role of Metformin as a mitochondrial inhibitor. Interestingly, recent evidence implies a prominent role for mitochondrial signaling not only in Clear Cell Renal Cell Carcinoma (64), but also in high-grade PCa (65). Potentially, angiogenesis inhibition could be more effective in patients suffering from PC when combined with adjuvants such as Metformin.

## Discussion

**Figure 1:**
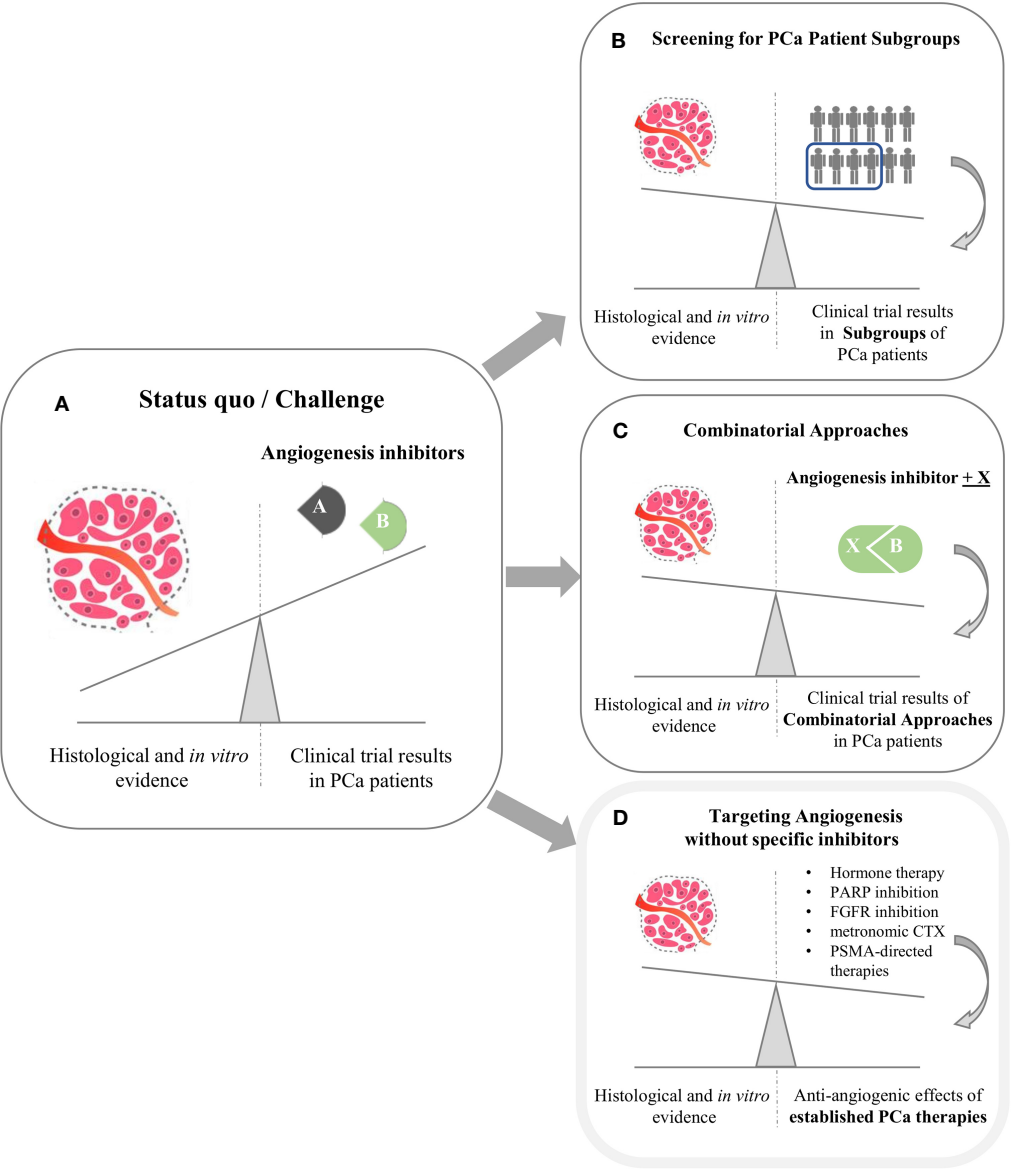
The clinical challenge of angiogenesis inhibition in Prostate Cancer (PCa). **(A)** Despite promising preclinical evidence from histopathological and *in vitro* analyses, phase III clinical trials with angiogenesis inhibitors failed to meet clinical endpoints. **(B, C)** Main strategies aiming to leverage the impact of angiogenesis inhibition are biomarker-aided identification of PC patient subgroups most susceptible towards anti-angiogenesis **(B)** and combinatorial approaches **(C)**. Moreover, several established PCa therapies partly exhibit anti-angiogenic effects as mode of action **(D)**.

From a histopathological and preclinical perspective, there is convincing evidence for a significant role of angiogenesis in PCa development and progression. For example, VEGFR2 was shown to mark PCa cases with a high risk of progression (30, 66). In addition, angiogenesis-related microRNAs such as let-7, miR-195 and miR-205 (67) are also deregulated and play prominent roles in PCa (68–70). However, no angiogenesis-specific inhibitor has met its clinical endpoint in phase III trials (see **Figure 1A**). Consequently, angiogenesis inhibitors currently do not play a role in PCa treatment guidelines. As shown by our database search on clinicaltrials.gov, several clinical trials are currently recruiting patients with PCa to address the discrepancy between promising preclinical findings and sobering clinical trial results.

### Current Therapeutic Strategies to Narrow the Gap Between Bench and Bedside

As illustrated in **Figure 1**, two main strategies aim to establish therapeutic anti-angiogenesis in patients with PCa. Within the first strategic approach, clinicians are searching for PCa subgroups most susceptible towards angiogenesis inhibition (**Figure 1B**). It is tempting to assume that targeting tumor neovascularization could be more efficient when used early in the course of disease (71) in order to prevent metastases (44, 72). In line with this assumption, clinicians examine effects in PCa subgroups other than mCRPC. Specifically, SPARC investigates Cabozantinib in a neoadjuvant setting. PCa patients suffering from biochemical recurrence are currently recruited for the BLAST trial, which investigates the JAK/FLT3 inhibitor Pacritinib. Moreover, the CABIOS trial recruits CSPC patients receiving Cabozantinib, Abiraterone and Nivolumab (thereby also representing the second strategic approach of combinatorial therapies). Up to now, neither predictive nor response biomarkers have been established to stratify PCa patients regarding anti-angiogenic therapy (18, 26). Of note, most biomarker-driven trials trying to meet the needs are not PCa-specific. Recruiting patients suffering from advanced cancer, the MATCH screening trial constitutes a biomarker-driven basket study for various compounds including Sunitinib. In a similar setting, SMMART investigates compounds such as Bevacizumab, Cabozantinib, Sorafenib and Sunitinib.

As a second strategic approach to narrow the gap between bench and bedside (**Figure 1C**), clinicians and researchers combine angiogenesis inhibitors with other established cancer compounds. Most of the respective trials identified by our search teamed angiogenesis inhibitors with immune checkpoint inhibitors (ICI) – e. g. Cabozantinib and Atezolizumab (CONTACT-02 trial). However, the primary rationale of these approaches is not to establish anti-angiogenesis as a treatment option for PCa, but to break therapy resistance towards ICI (73–75).

### BRCA in Metastatic Prostate Cancer - Recommendations and Perspectives

As a second bullet point to envision next steps narrowing the gap between the bench and bedside, it is important to highlight that genetic alterations of BRCA2 and BRCA1 occur in metastatic PCa with a frequency of 13% and 5.3% for the somatic component, and 0.3% and 0.9% for the germline component, respectively (76, 77). Germline mutations in BRCA2 are associated with pathways also related to VEGF signaling (78). Thus, phase II and III studies investigating effect on PFS and ORR in mCRPC hold promise to further elucidate the complex relationship of disease biology, since genomic alterations and several genes are screened (**Table 2**). TRITON2 and GALAHAD studies showed objectives and PSA responses in patients with BRCA1/2 alterations employing Rucaparib and Niraparib, respectively (79, 80). Nonetheless, the Profound trial testing Olaparib, confirmed that BRCA2 is the most frequently altered gene and with BRCA1 and ATM genes allowed to reach a radiographic PFS improvement of Olaparib treated over control (HR.34 P<.0001, CI.25-.47). Those results are remarkable since checkpoint inhibitors may have limited efficacy in PCa as single agents; thus, combination approaches are being examined to potentially improve their efficacy in this as in other urological diseases (30, 44). The hypothetical synergism between PARP inhibitors and ICI is centered on evidence that DNA damage resulting from PARP inhibition triggers the cGAS-STING pathway (81), which consequently boosts the interferon signaling, leading to enhanced immunogenicity (82). There is also rationale for an additive effect in cancers with high microsatellite instability (MSI) and BRCA mutations (83). Moreover, cancers with CDK12 mutations are often sensitive to PARP inhibitors - and preclinical and biological data from patients with PCa showed that CDK12 inactivation is related to increased burden of neoantigens, which can in turn enhance the immunogenicity (84). ICI hold anti-mCRPC activity potential in high degree of MSI. Indeed, the KEYNOTE-365 trial comparing Pembrolizumab plus Olaparib in biomarker-unstratified mCRPC subjects after prior taxane-based regimen uncovered that 36.6% of individuals obtained a PSA response (85). The KEYLYNK-010 phase III study has been designed to deeper elucidate the combination of Pembrolizumab plus Olaparib in patients with mCRPC in a biomarker-unselected population after progression on androgen-deprivation therapy and androgen receptor signaling inhibitor (86). In line with this, Nivolumab plus Rucaparib in the phase II CheckMate 9KD trial focusing on mCRPC revealed that best response rates were among BRCA2 mutated cases and that the combination was not efficient in individuals without homologous recombination mutations (87). Statistically powered studies aiming to corroborate these hypothesis-generating results are needed. Nonetheless, based on the available data, the FDA approved both Niraparib and Rucaparib as well as Olaparib in May 2020 (88). Nonetheless, EMA approved Olaparib for the treatment of patients with mCRPC and BRCA1/2 mutations, either germline or somatic after progression following a prior line including a hormonal agent, based on the results published by Hussain M. et al. (89). Collectively, the BRCA mutational status assessment in mCRPC is not merely a predictor of response to PARP inhibition, but is rather a biomarker of aggressiveness and therefore can sketch a disease phenotype for whom additional biomarker might be added (90). Indeed, BRCA status might also predict a decreased taxane sensitivity compared to Abiraterone and Enzalutamide, nonetheless confirmatory trials are also needed.

**Table 2:** Trials screening genes involved in prostate cancer (PCa) registered within clinicaltrials.gov database (December 2021). See text for details.

	PROFOUND	TRITON 2	GALAHAD
Drug	Olaparib 300 mg bid	Rucaparib 600 mg bid	Niraparib 300 mg qd
Study design	Phase III	Phase II	Phase II
Population	mCRPC progression to ARSI	mCRPC progression to ARSI and taxane	mCRPC progression to ARSI and taxane
Primary objective	rPFS in pts with alterations in ATM, BRCA1, BRCA2	ORR and PSA response (≥50% decline) in pts with DDR alterations	ORR in patients with bi-allelic BRCA1/2 alterations
Specimen tested	Tumor tissue central	Plasma or tumor tissue central/local	Plasma central
Test used	FoundationOne^®^	FoundationOne^®^ FoundationACT^®^ Local	Resolution-HRD^®^
Genes screened	*ATM, BARD1, BRCA1, BRCA2, BRIP1, CDK12, CHEK1, CHEK2, FANCL, PALB2, PPP2RA, RAD51B, RAD51C, RAD51D, RAD54L*	*ATM, BARD1, BRCA1, BRCA2, BRIP1, CDK12, CHEK2, FANCA, NBN, PALB2, RAD51, RAD51B, RAD51C, RAD51D, RAD54L*	*ATM, BRCA1, BRCA2, BRIP1, CHEK2, FANCA, HDAC2, PALB2*
Genomic alteration required	Mono- and Bi- allelic alterations in DDR genes	Mono- and Bi- allelic alterations in DDR genes	Bi- allelic alterations in DDR genes

### Targeting Angiogenesis Without Specific Inhibitors – Established and Evolving Therapies

While our database search on clinicaltrials.gov revealed a limited number of studies with specific inhibitors of angiogenesis, a plethora of trials investigated compounds such as antiandrogens, PARP inhibitors and PSMA-directed agents. At first sight, these approaches might not appear tightly related to tumor angiogenesis. Yet, recent findings imply that all these strategies obtain a significant anti-angiogenic component. Regarding AR-related signaling, a growing amount of literature investigates the complex crosstalk with VEGF-mediated pathways in cancer (91). As mentioned, for PARP inhibitors such as Olaparib, an anti-angiogenic effect besides an anti-mCRPC is widely accepted (14, 92, 93). Moreover, FGF (Fibroblast Growth Factor) and its receptors (FGFRs) play prominent pro-angiogenic roles in several malignancies, including PCa (94, 95). Consequently, the FGFR inhibitor Erdafitinib is currently investigated in patients with CRPC as a single drug (NCT04754425) and combined with Abiraterone or Enzalutamide in patients with CRPC (NCT03999515).

Metronomic (low-dose) chemotherapy is another well-described therapeutic strategy to target tumor-associated neo-vasculature in various cancer entities. Frequent and regular administration of chemotherapeutic agents at doses constituting a fraction of the MTD (maximum tolerated dose) was shown to have substantial therapeutic effects – especially on tumor endothelium. Moreover, these regimens frequently exhibited favorable toxicity profiles (96, 97). For PCa, clinical evidence highlights the potential of metronomic therapies especially in mCRPC: studies investigated metronomic Cyclophosphamide in combination with Docetaxel (98) or in heavily pretreated patients after Docetaxel or Abiraterone/Enzalutamide (99–102) – showing effectiveness and good tolerability. In addition, researchers examined the efficacy of metronomic application of Vinorelbine (103) and metronomic Cyclophosphamide, Celecoxib and Dexamethasone in patients suffering from mCRPC (104). Interestingly, metronomic Cyclophosphamide application also induced an immune reaction (in terms of T cell reactivation) in patients with biochemical recurrence (105). Although the mode of action of metronomic therapies is not completely understood, a recent study identified key genes which were associated with (metronomic) Topotecan dosing in PCa cell lines (106).

Regarding PSMA, receptor expression not only exists on the surface of PCa cells. Instead, tumor-associated endothelium frequently displays robust levels of PSMA in various cancer entities (107–109). Future research must show the impact of targeting PSMA in terms of anti-angiogenic activity – for PCa but also for other entities with PSMA-positive tumor endothelium. Given the rationale of adding angiogenesis inhibitors to ICI in order to break resistance towards immune-based approaches (73–75), it also appears tempting to assume that targeting PSMA could have an impact on the immunogenicity of PCa.

In a nutshell: While specific angiogenesis inhibitors currently do not have an established role in PCa, targeting tumor angiogenesis and tumor-associated blood vessels probably is part of established PCa therapies – especially regarding PSMA-directed approaches.

## Conclusion

Targeting angiogenesis with specific inhibitors unfortunately has failed to impact OS in patients with mCRPC despite promising early data – and despite convincing clinical activity in several other malignancies. This discrepancy highlights the importance of the microenvironment niche, as PCa is characterized by substantial inter- and intra-patient heterogeneity and adaptive biology. Therapeutic strategies to overcome this challenge include biomarker-guided screening for patient subgroups most likely to benefit from anti-angiogenesis. Moreover, several trials investigate combinatorial approaches. Beyond specific angiogenesis inhibitors, approved compounds such as antiandrogens, PARP inhibitors and PSMA-targeting approaches probably also have a substantial anti-angiogenic impact in PCa biology.

## Author Contributions

Conceptualization: AS and MK. Methodology: AS and MK. Writing – draft preparation: AS, CK, and MK. Writing – review and editing: AS, CK, and MK. All authors contributed to the article and approved the submitted version.

## Funding

This project was supported in part by the Apulian Regional Project Medicina di Precisione to A.G.S. Moreover, M.K. was funded by a personal grant from Else-Kröner-Foundation (Else Kröner Integrative Clinician Scientist College for Translational Immunology, University Hospital Würzburg, Germany). This publication was supported by the Open Access Publication Fund of the University of Würzburg.

## Conflict of Interest

The authors declare that the research was conducted in the absence of any commercial or financial relationships that could be construed as a potential conflict of interest.

## Publisher’s Note

All claims expressed in this article are solely those of the authors and do not necessarily represent those of their affiliated organizations, or those of the publisher, the editors and the reviewers. Any product that may be evaluated in this article, or claim that may be made by its manufacturer, is not guaranteed or endorsed by the publisher.
